# *InfectiousDiseases ofPoverty*, the first five years

**DOI:** 10.1186/s40249-017-0310-6

**Published:** 2017-05-04

**Authors:** Wei Wang, Jin Chen, Hui-Feng Sheng, Na-Na Wang, Pin Yang, Xiao-Nong Zhou, Robert Bergquist

**Affiliations:** 1 Key Laboratory of National Health and Family Planning Commission on Parasitic Disease Control and Prevention, No. 117 Yangxiang, Meiyuan, Wuxi City, Jiangsu Province 214064 China; 2 Jiangsu Provincial Key Laboratory on Parasites and Vector Control Technology, No. 117 Yangxiang, Meiyuan, Wuxi City, Jiangsu Province 214064 China; 3 Jiangsu Institute of Parasitic Diseases, No. 117 Yangxiang, Meiyuan, Wuxi City, Jiangsu Province 214064 China; 4 School of Public Health, Fujian Medical University, No. 88 Jiaotong Road, Fuzhou City, Fujian Province 350004 China; 5 National Institute of Parasitic Diseases, Chinese Center for Disease Control and Prevention, No. 207 Rui Jin Er Road, Shanghai, 200025 China; 6 WHO Collaborating Center for Tropical Diseases, No. 207 Rui Jin Er Road, Shanghai, 200025 China; 7 Key Laboratory of Parasite and Vector Biology, Ministry of Health, No. 207 Rui Jin Er Road, Shanghai, 200025 China; 8 Editorial Office of Chinese Journal of Clinical Research, No. 57 Shanxi Road, Nanjing City, Jiangsu Province 210009 China; 9 Ingerod, Brastad, Sweden

**Keywords:** Infectious diseases of poverty, Impact, Impact factor, Bibliometric analysis, Content analysis

## Abstract

Although the focus in the area of health research may be shifting from infectious to non-communicable diseases, the infectious diseases of poverty remain a major burden of disease of global health concern. A global platform to communicate and share the research on these diseases is needed to facilitate the translation of knowledge into effective approaches and tools for their elimination. Based on the “One health, One world” mission, a new, open-access journal, *Infectious Diseases of Poverty* (*IDP*), was launched by BioMed Central in partnership with the National Institute of Parasitic Diseases (NIPD), Chinese Center for Disease Control and Prevention (China CDC) on October 25, 2012. Its aim is to identify and assess research and information gaps that hinder progress towards new interventions for a particular public health problem in the developing world. From the inaugural *IDP* issue of October 25, 2012, a total of 256 manuscripts have been published over the following five years. Apart from a small number of editorials, opinions, commentaries and letters to the editor, the predominant types of publications are research articles (69.5%) and scoping reviews (21.5%). A total of 1 081 contributing authors divided between 323 affiliations across 68 countries, territories and regions produced these 256 publications. The journal is indexed in major international biomedical databases, including Web of Science, PubMed, Scopus and Embase. In 2015, it was assigned its first impact factor (4.11), which is now 2.13. During the past five years, *IDP* has received manuscripts from 90 countries, territories and regions across six continents with an annual acceptance rate of all contributions maintained at less than 40%. Content analysis shows that neglected tropical diseases (NTDs), followed by the “Big Three” (HIV/AIDS, malaria and tuberculosis) and infectious diseases in general comprise 88% of all publications. In addition, a series of 10 thematic issues, covering 118 publications in all, was published as separate parts of the first five volumes. These publications were cited 975 times, which equals an average of 8.3 times per publication. The current challenge is to identify cutting-edge research topics and attract and to publish first-rate publications leading to increasing importance and impact of the journal in its field.

**Electronic supplementary material:**

The online version of this article (doi:10.1186/s40249-017-0310-6) contains supplementary material, which is available to authorized users.

## Multilingual abstracts

Please see Additional file 1 for translations of the abstract into the six official languages of the United Nations.

## Background

The global burden of disease today is less due to infectious diseases than non-communicable diseases, with chronic disorders such as heart disease, stroke and cancer currently being the leading causes of morbidity and mortality worldwide [1]. However, infections remain a major global health concern, particularly in the developing world [2], where the human immunodeficiency virus and the acquired immune deficiency syndrome (HIV/AIDS), viral hepatitis, tuberculosis and malaria, still kill millions of people around the globe every year [3, 4]. Parasitic diseases, e.g., malaria and schistosomiasis, do not always remain limited to their age-old geographic distributions complicating control efforts and challenging the progress towards their elimination [5–8]. Additionally, emerging and re-emerging infectious diseases like the severe acute respiratory syndrome (SARS), influenza, Ebola virus disease (EVD), dengue, Middle East respiratory syndrome (MERS) and Zika virus disease threaten human health and global security [9–18].

Infectious diseases are inextricably linked to poverty in a vicious cycle [19]. These diseases, characterized by high morbidity and mortality that mainly occur in resource-limited areas, belong by definition to the group of diseases that are more prevalent among poor and vulnerable populations [20]. They rank within the top ten ailments with respect to years lost due to ill-health, disability or early death, expressed as disability-adjusted life years (DALYs) [3]. In a global perspective, this leads to huge economic losses, both for the individual and society, and a global platform to communicate and share the research on the infectious diseases of poverty was felt to be needed to facilitate the translation of knowledge into effective approaches and tools for the elimination of these diseases.

On October 25, 2012, *Infectious Diseases of Poverty* (*IDP*) was launched as a new, open-access (OA) journal on infectious diseases by BioMed Central, in partnership with the National Institute of Parasitic Diseases (NIPD), Chinese Center for Disease Control and Prevention (CDC). Its preferential aims are to identify and assess research and information gaps that hinder progress towards new interventions for public health problems, in particular those connected with poverty in the developing world. Based on the “One health, One world” mission mentioned in the *Global Report on Research for the Infectious Diseases of Poverty* [21], the journal publishes work on topics and approaches that address essential public health questions related to this issue. Along with the actions proposed by this report, *IDP,* aims to achieve the goal of improving research capacity and create a better environment for the research on the infectious diseases of poverty [19, 22].

The purpose of this article is to review the publication activities of *IDP* in its first five years by comparing the record with other journals with a similar focus in order to understand the current trends of research in publications on infectious diseases.

## Methods

We performed an in-depth bibliometric analysis of all publications during the first five volumes covering the period from October 25, 2012 to October 25, 2016 through a joint search using the publication name “*Infectious Diseases of Poverty*” in PubMed, Web of Science (WOS) (formerly ISI Web of Knowledge) and the journal’s website. We also compared with two other journals in this area, *International Journal of Infectious Diseases* and *BMC Infectious Diseases*.

The publication data and metrics of *IDP*, *International Journal of Infectious Diseases* and *BMC Infectious Diseases* were collected using these journals’ homepages, submission systems and the WOS Citation Database as of 20th February, 2017. We used ordinary descriptive statistics to analyse the performance of *IDP* during its first five years supported by BioMed Central’s databases [23].

## Results

### Editorial board

*IDP* is currently managed by an Editor-in-Chief and two managing editors, one based in China and the other in France. They perform the bulk of the day-to-day activities, including communication with authors, choosing and contacting referees for peer review, and making the final decisions with regard to publication or rejection of manuscripts. This core team is supported by five Deputy Editors and 13 Associate Editors from Asia, Africa, Europe, North America, South America and Oceania.

To assure quality and impact from the start, *IDP* appointed an international editorial board of well-known scientists with expertise within the fields of infectious diseases, parasitic diseases, social sciences and economy. The current editorial board consists of 31 members, based in UK (*n* = 4), USA (*n* = 3), China (*n* = 3), Australia (*n* = 3), Switzerland (*n* = 3), Brazil (*n* = 2), Cameroon (*n* = 2), India (*n* = 2), Nigeria (*n* = 1), Japan (*n* = 1), Ghana (*n* = 1), Belgium (*n* = 1), Thailand (*n* = 1), Greece (*n* = 1), Democratic Republic of Congo (*n* = 1), South Africa (*n* = 1), Kenya (*n* = 1) and Senegal (*n* = 1).

### Bibliometric analysis

**Fig. 1:**
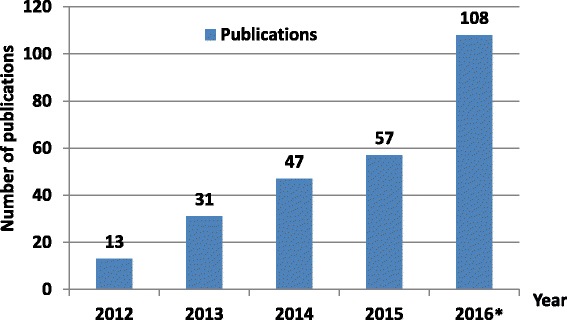
Annual number of submissions and publications in *IDP* between October 2012 and October 2016

**Fig. 2:**
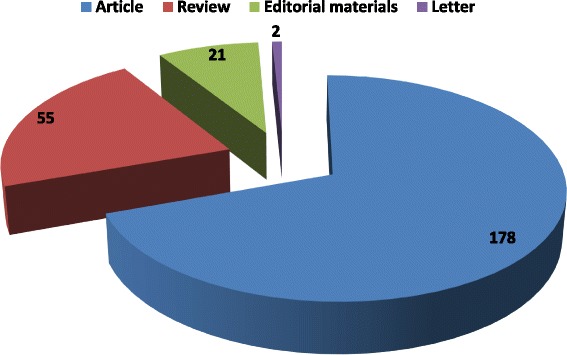
Types of publications in *IDP* between October 2012 and October 2016

From the inaugural issue of October, 2012 up to and including October, 2016, a total of 256 manuscripts were published in *IDP*. The annual publication record is presented in Fig. 1. Out of all publications, research articles (69.5%) and scoping reviews (21.5%) dominated with the remaining 7 publication being editorials, opinion articles and letters to the editor (Fig. 2). A total of 1 081 contributing authors, divided between 323 affiliations across 68 countries, territories and, regions produced these 256 publications during the five-year period (Tables 1, 2 and 3). The publications included 53 countries, territories and regions from the developing world (78%) [24].

**Table 1:** The 10 strongest contributors by affiliation in the 256 publications in the first five volumes of *IDP*

Affiliation	Number of publications
Chinese Center for Disease Control and Prevention	47
Duke University	16
WHO	16
Chinese University of Hong Kong	11
WHO Collaborating Center for Malaria, Schistosomiasis, and Filariasis	10
Duke Kunshan University	9
Swiss Tropical Public Health Institute	9
University of Basel	9
The Aga Khan University	8
Fudan University	8

**Table 2:** The 10 strongest contributors by country in the 256 publications in the first five volumes of *IDP*

Country	Number of publications
People’s Republic of China	115
United States of America	48
United Kingdom	32
Switzerland	24
Canada	17
Nigeria	15
Australia	14
France	14
India	14
Pakistan	13

**Table 3:** The most 10 contributing authors in the 256 publications in the first five volumes of *IDP* (from the inaugural issue to October 25, 2016)

Author	Number of publications
Xiao-Nong Zhou	18
Zulfiqar A Bhutta	8
Jai K Das	8
Rehana A Salam	8
Ernest Tambo	8
Qian Long	7
Shenglan Tang	7
Jun-Hu Chen	6
Zohra S Lassi	6
Robert Bergquist/Henry Lucas/Guo-Jing Yang	5

### Impact

**Fig. 3:**
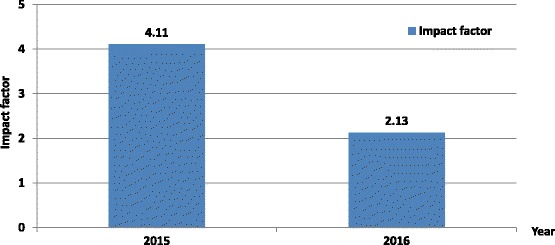
Impact factor of *IDP* in 2015 and 2016

The journal is indexed in major international biomedical databases, including WOS, Science Citation Index Expanded, MEDLINE, DOAJ, PubMed, Scopus and Embase. In 2015, it was assigned the first impact factor (4.11), which is now 2.13 (Fig. 3).

### The citation record

Up to 20th February 2017, the 256 publications in the first five *IDP* volumes were cited 1 187 times in total, i.e. a mean citation of 4.64 times per paper. Table 4 presents the 10 most highly cited publications, which includes five reviews, two editorials, one opinion article, one research article and one letter to the editor. Interestingly, the most highly cited publication in *IDP* is an editorial published in 2013 [25], which may be explained by the fact that it dealt with surveillance and response defining this approach as a research priority during the stage moving towards elimination of tropical diseases, which received much global attention [26–30]. The importance and significance of the surveillance and response approach are also highlighted by two other highly cited publications in *IDP* [26, 31]. We refer here to two reviews, one discussing gap analysis of the Chinese three major tropical diseases (schistosomiasis, malaria and echinococcosis), and the other assessing the morbidity due to schistosomiasis japonica in China [32, 33]. In addition, an editorial opinion focusing on the Chinese schistosomiasis control and health systems and a research article reporting on *Babesia microti* and *Plasmodium* co-infections along the China-Myanmar border were also commonly cited [34, 35]. Among three other publications, which have been cited 26 times and more, are two reviews describing global epidemiology: the burden of infectious diseases of poverty [2] and that of clonorchiasis [36], together with an editorial introducing the mission, aims and scope of *IDP* [37].

**Table 4:** The 10 most highly cited papers published in the first five volumes of *IDP* (from the inaugural issue to October 25, 2016)^a^

Title	Corresponding author	Corresponding author’s affiliation	Publication year	Total citation in WOS	Type of publication
Elimination of tropical disease through surveillance and response	Xiao-Nong Zhou	National Institute of Parasitic Diseases, Chinese Center for Disease Control and Prevention; WHO Collaborating Centre for Malaria, Schistosomiasis and Filariasis; Key Laboratory of Parasite and Vector Biology, Ministry of Health, People’s Republic of China	2013	46	Editorial
Research gaps for three main tropical diseases in the People’s Republic of China	Hao-Bing Zhang	National Institute of Parasitic Disease, Chinese Center for Disease Control and Prevention; WHO Collaborating Centre on Malaria, Schisostomiasis and Filariasis; Key Laboratory of Parasite and Vector Biology, Ministry of Health, People’s Republic of China	2013	42	Scoping review
Surveillance-response systems: the key to elimination of tropical diseases	Marcel Tanner or Xiao-Nong Zhou	Department of Epidemiology and Public Health, Swiss Tropical and Public Health Institute and University of Basel, Switzerland (Marcel Tanner) or National Institute of Parasitic Diseases, Chinese Center for Disease Control and Prevention; WHO Collaborating Centre for Malaria, Schistosomiasis and Filariasis; Key Laboratory of Parasite and Vector Biology, Ministry of Health, People’s Republic of China (Xiao-Nong Zhou)	2014	40	Scoping review
Schistosomiasis control and the health system in PR China	Shenglan Tang	Duke Global Health Institute, Duke University, USA.	2012	40	Opinion
Global burden, distribution, and interventions for infectious diseases of poverty	Zulfiqar A Bhutta	Center of Excellence in Women & Child Health, The Aga Khan University, Karachi, Pakistan; Center for Global Child Health Hospital for Sick Children, Toronto, Canada.	2014	32	Scoping review
Need of surveillance response systems to combat Ebola outbreaks and other emerging infectious diseases in African countries	Ernest Tambo	Sydney Brenner Institute for Molecular Bioscience, Wits 21st Century Institute, Faculty of Health Sciences, University of the Witwatersrand; Center for Sustainable Malaria Control, Department of Biochemistry, Faculty of Natural and Agricultural Sciences, University of Pretoria, South Africa; National Institute of Parasitic Diseases, Chinese Center for Disease Control and Prevention, and the WHO Collaborating Centre on Malaria, Schisostomiasis and Filariasis, PR China.	2014	29	Letter to the Editor
The global epidemiology of clonorchiasis and its relation with cholangiocarcinoma	Men-Bao Qian	National Institute of Parasitic Diseases, Chinese Center for Disease Control and Prevention; WHO Collaborative Center for Malaria, Schistosomiasis and Filariasis; Key Laboratory of Parasite and Vector Biology, Ministry of Health, People’s Republic of China.	2012	28	Scoping review
Assessment of morbidity due to *Schistosoma japonicum* infection in China	Ming-Gang Chen	National Institute of Parasitic Diseases, Chinese Center for Disease Control and Prevention; WHO Collaborative Center for Malaria, Schistosomiasis and Filariasis, People’s Republic of China.	2014	26	Scoping review
Prioritizing research for “One health - One world”	Xiao-Nong Zhou	National Institute of Parasitic Diseases, Chinese Center for Disease Control and Prevention, People’s Republic of China.	2012	26	Editorial
Co-infections with *Babesia microti* and *Plasmodium* parasites along the China-Myanmar border	Wei Hu	National Institute of Parasitic Diseases, Chinese Center for Disease Control and Prevention; WHO Collaborating Centre for Malaria, Schistosomiasis and Filariasis; Key Laboratory of Parasite & Vector Biology Ministry of Health; Department of Microbiology and Microbial Engineering, School of Life Science, Fudan University, China.	2013	24	Research article

^a^ Citation was calculated until February 20, 2017

### Downloads and reads

The number of articles of the 256 publications accessed through Internet reached a total of 1 174 098 accesses up to 20th February, 2017, with a mean of 4 586.32 times per publication. Table 5 summarizes the five most highly accessed publications, which have been accessed more than 12 000 times. They include three research articles, one focusing on the interplay between infectious disease emergence and global climate change [38], one dealing with the risk factors of malaria among pregnant women in Nigeria [39] and one discussing the reaction of Chinese social media to the outbreaks caused by the MERS coronavirus and avian influenza A (H7N9) [40]. Two reviews, one on tuberculosis [40] and the other on malaria and other vector-borne protozoan diseases [41] were also often downloaded. Interestingly, the most highly accessed publication is a research article exploring the potential correlation between the emergence of infectious diseases and global climate change, perhaps explained by the currently worldwide focus on a large number of emerging and re-emerging infectious diseases in this context [42, 43].

**Table 5:** The 5 most highly accessed papers published in the first five volumes of *IDP* (from the inaugural issue to October 25, 2016)^a^

Title	Corresponding author	Corresponding author’s affiliation	Publication year	Total accesses	Type of publication
Infectious disease emergence and global change: thinking systemically in a shrinking world	Colin D Butler	National Centre for Epidemiology and Population Health College of Medicine Biology and Environment, Australian National University, Australia.	2012	24 570	Research article
Community based interventions for the prevention and control of tuberculosis	Zulfiqar A Bhutta	Center of Excellence in Women & Child Health, The Aga Khan University, Karachi, Pakistan; Center for Global Child Health Hospital for Sick Children, Toronto, Canada.	2014	20 998	Scoping review
Factors associated with risk of malaria infection among pregnant women in Lagos, Nigeria	Wellington A Oyibo	ANDI Centre of Excellence for Malaria Diagnosis, International Malaria, Microscopy Training and RDT QA Programme, WHO/TDR/FIND Malaria Specimen Bank Site, Department of Medical Microbiology and Parasitology, College of Medicine, University of Lagos, Nigeria.	2013	14 839	Research article
Control of malaria and other vector-borne protozoan diseases in the tropics: enduring challenges despite considerable progress and achievements	Denis Zofou	Biotechnology Unit, Faculty of Science, University of Buea, Cameroon.	2014	12 539	Scoping review
Chinese social media reaction to the MERS-CoV and avian influenza A(H7N9) outbreaks	Isaac Chun-Hai Fung	Department of Epidemiology, Jiann-Ping Hsu College of Public Health, Georgia Southern University, USA.	2013	12 870	Research article

^a^ Access was calculated until February 20, 2017

### Publication quality and origins

**Fig. 4:**
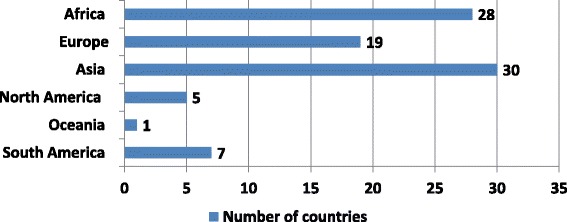
Country origin of all manuscripts submitted to *IDP* from October 2012 to October 2016

**Fig. 5:**
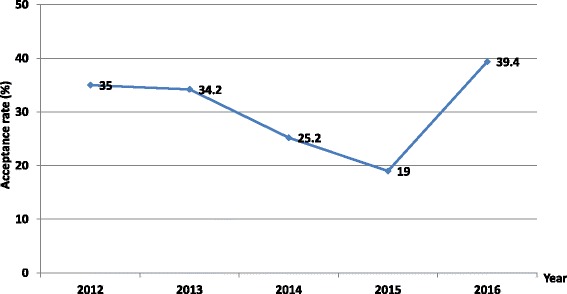
Annual acceptance rate of all manuscripts submitted to *IDP* from October 2012 to October 2016

During the 5-year period investigated, countries *IDP* received manuscripts from 90 countries, territories and regions across six continents (Fig. 4) that included 61 from the developing world (68%) [24]. The annual acceptance rate of all submitted manuscripts received was maintained at less than 40% throughout the period analysed (Fig. 5).

### Diseases investigated

**Fig. 6:**
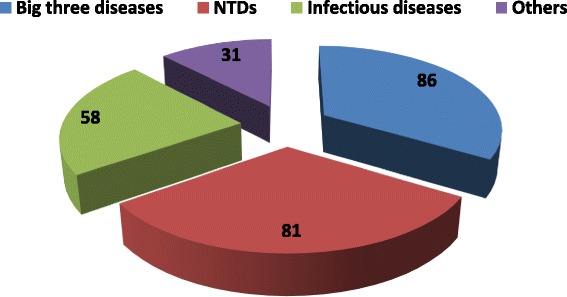
Pie chart showing the diseases featured in the 256 publications in *IDP* from October 2012 to October 2016

Content analysis showed that neglected tropical diseases (NTDs), followed by the “Big Three” (HIV/AIDS, malaria and tuberculosis) and other infectious diseases, were in the focus of the 256 publications, which consisted of 88% of total publications (Fig. 6). *IDP* is dedicated to communicate global health concerns on finding ways for poverty alleviation and to publish papers dealing with the following topics: (1) approaches addressing essential public health questions related to infectious diseases of poverty; (2) multi-disciplinary concerns of infectious disease of poverty, such as the biology of pathogens, vectors, diagnosis, surveillance and response, treatment and case management, epidemiology including ecohealth issues and modelling, zoonoses and animal reservoirs, control strategies and implementation of new technologies; and (3) trans-disciplinary or multi-sectoral effects involving health systems, environmental management and innovative technology.

### Role of the thematic series

A total of 10 thematic series were published in the first five volumes of *IDP* with 1 – 3 issues appearing each year (Table 6). The thematic series were issued to allow the journal to remain in the frontline in the field of infectious diseases of poverty underpinning international readership. The series covered a variety of diseases, including EVD, malaria, schistosomiasis, tuberculosis and other infectious diseases, and they were particularly planned to address important issues and approaches, e.g., surveillance and response, interplay between environmental change and emerging infectious diseases (EIDs), insecticide resistance, etc. A total of 118 research articles, accounting for 46% of the contents published, were together cited 975 times. This means an average citation rate of 8.3 times per publication, which is significantly higher than the mean citation (4.64) of the all the 256 papers published since the journal was launched.

**Table 6:** Thematic series published in the first five volumes of *IDP* (from the inaugural issue to October 25, 2016)^a^

Thematic series	Year of publication	Number of publications in the thematic series	Total citation	Citation per publication
Health Systems Research for Infectious Diseases of Poverty	2012	12	203	16.9
Surveillance and Response to Infectious Diseases of Poverty	2013	23	278	12.1
Co-infection and Syndemics	2013	16	121	7.6
Historical Development of Medical Parasitology in China	2013	8	71	8.9
EcoHealth and EIDs - Dynamics between environmental change, development, and EIDs in Asia	2014	17	120	7.1
Community-Based Interventions for the Prevention and Control of Infectious Diseases of Poverty	2014	8	72	9
Ebola outbreaks and community-based surveillance response systems	2014	11	66	6
Insecticide Resistance in Vectors	2015	6	4	0.7
Improving Access to and Affordability of Healthcare for TB Patients in China	2016	10	13	1.3
Malaria and migration in the Greater Mekong Subregion	2016	7	27	3.9

^a^ Citation was calculated until February 20, 2017

### Comparison with other journals publishing in similar fields

**Fig. 7:**
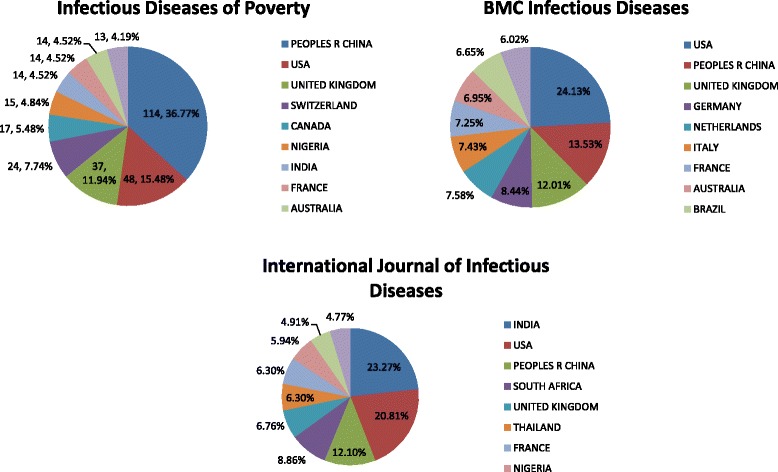
The top 10 contributing countries, as determined by authors’ affiliations, in the three journals from 2012 to 2016

Unlike *International Journal of Infectious Diseases* and *BMC Infectious Diseases*, two international, peer-reviewed journals in the field of infectious diseases that have attracted global contributions, *IDP* has focused on developing countries, in particular in Africa and Asia. Figure 7 shows the 10 countries that contributed the most to *IDP, International Journal of Infectious Diseases* and *BMC Infectious Diseases*. The 10 countries that contributed more than others to *BMC Infectious Diseases* included eight industrialized countries in North America, Europe and Oceania and two developing countries: Peoples’ Republic of China and Brazil. In the case of *International Journal of Infectious Diseases,* four industrialized countries from North America and Europe and four developing countries from Asia and Africa were included, while the 10 *IDP* top-contributing countries included six industrialized countries in North America, Europe, and Oceania and four developing countries in Asia and Africa. The difference in the distribution of contributing countries may be explained by the slightly different scopes and disease spectra in the three journals.

## Discussion

The World’s currently biggest donor for scientific research, the Bill and Melinda Gates Foundation (BMGF) demands that the outcomes of what it supports must be freely available to all. This is set to change scientific research publications at its core, since this requirement bars publication in ‘premier-league’ journals such as *Nature* and *Science*. Increasingly, scientific journals are accepting the OA mandate and *IDP* wisely decided to follow suit when its first volume appeared more than five years ago. As an international, peer-reviewed OA journal, *IDP* has its own specific characters as follows:

1. The journal provides the abstracts in the six official languages of the United Nations (UN), which enables clear and convenient communication of public health concerns related to the infectious diseases of poverty [19].
2. Fast-track publication is provided for articles of exceptional public health importance and urgency with online publication completed within a month of submission.
3. The journal focuses on publication of original and empirical trans-disciplinary research for the control of infectious diseases which predominantly affect poor populations.

Free preprint repository, *bioRxiv* (http://biorxiv.org/), is another revolution quietly taking place and gradually found more and more useful for scientists as it establishes priority and exposure, while, on the other hand, there is no peer-review. This important development must be taken under consideration in the publication world and scientific journals, including, *IDP*, need to find a way to participate in this expansion of how new findings are brought into the public sphere.

In 2014, a thematic series issue entitled “EcoHealth and EIDs - dynamics between environmental change, development and EIDs in Asia”, which was supported by the International Development Research Centre (IDRC) of Canada and published with the aim to showcase and encourage trans-disciplinary EcoHealth research in Asia and beyond. The 17 publications in this thematic series were cited 120 times, with a mean of 7.1 times per publication. It attracted 38 352 downloads and reads in total, with a mean of 2 256 per publication.

Since the launch and continuing, *IDP* aims to identify and assess research and information gaps that hinder progress towards new interventions for the particular public health problem of poverty and infections in the developing world. In its first five volumes, the journal published studies from 53 developing economies (34%) and attracted contributions from 61 developing economies (39%); however, there are still more than 60% of the global developing economies that have not yet contributed a paper to the journal. Further work to attract manuscripts from the developing world is urgently needed. In addition, 31% (284/917) of all manuscripts in the first five years were submitted by African scientists; however, even if only slightly more than 33% (95/284) of these were finally published, this indicates a strong African interest in publishing with *IDP* as well as a trend of improving quality of research conducted in Africa.

The journal was assigned a first impact factor of 4.11 in 2015; however, this metric decreased to 2.13 in 2016, which can be explained by the large rise in the number of annual publications achieved already the following year. The huge increase in manuscripts submitted to *IDP* translates into a remarkable rise in the publications in the journal, resulting in a challenge to increase of the journal impact factor. Further analysis showed that 5 of the 256 publications in the first five volumes had more than 30 citations, while 66 publications had none, including 50 in the single year of 2016. Publication of high-quality studies and ‘hot’ research topics are believed not only to build an international following, but also improve the journal’s impact factor.

The journal may seem less attractive for contributions pertaining to high-impact topics in infectious disease research compared to *International Journal of Infectious Diseases* and *BMC Infectious Diseases*, which may be explained by the fact that it is a relatively new journal, which has not yet garnered an adequate international following. Publication of ‘hot’ research topics and results changing the direction of research should be strived for and this can be achieved through invited manuscripts and thematic series. In the future, work attracting high-quality papers from Europe and USA in addition to papers from the developing world, should be strengthened.

During the past five years, *IDP* has been strengthening its academic impact; however, its international impact remains to be enhanced relative to other leading journals in infectious diseases. The following priority has been recommended by its editorial board:

1. Invitation of contributions from global health policy makers and high-impact scientists;
2. Publication of thematic series pertaining to global ‘hot’ topics in public health, emerging or re-emerging diseases and the outcome of international major projects on infectious diseases;
3. Publication of thematic series pertaining to health policy in collaboration with the Special Programme for Research and Training in Tropical Diseases (TDR) of the World Health Organization (WHO), which may facilitate the development of a global health policy and increase its impact.

In addition, it might be useful to mark the official WHO health days, as mandated by the World Health Assembly, e.g., World Health Day on 7 April; World TB Day on 24 March; World Malaria Day on 25 April; World Hepatitis Day on 28 July; and World AIDS Day on 1 December.

Finally, the question of a paper’s true value must be mentioned as it is at the heart of the collaboration between author(s) and journal. That question extends naturally to the question of what the true value of a scientific journal is. This question has been approached in various ways, not the least by awarding impact factors. This first attempt is still the most utilized metric for assessing a journal’s merit, but new developments are telling us that a faster and wider range of research-related metrics are needed. The number of clicks, downloads and reads are already providing valuable insights into how published content is being used and shared by fellow scientists. A multitude of commercial companies are already active in this field, for example, Altmetric (https://www.altmetric.com/), Plum Analytics (http://plumanalytics.com/) and ResearchGate (https://www.researchgate.net/). Meta (http://www.meta.com) has gone one step further by using algorithms recognizing the unique features characterizing widely cited papers, and the company claims that this can predict a paper’s future impact before its publication. More than 26 million papers and 14 million researchers have already been profiled by the company (https://en.wikipedia.org/wiki/Meta) to support this claim.

## Conclusions

*IDP* has achieved its preliminary goal to become a platform for the identification of research and information gaps that hinder progress towards new interventions for public health problem connected to poverty in the developing world. However, there is still a long way to go to further advance research and evidence-building based on improved public health interventions in poor settings. It is believed that *IDP* can significantly facilitate progress towards the elimination of infectious diseases related to poverty. In this process, *IDP* will not only be judged by the quality of the articles published, but also regarding how it fits into the new world of merit assessment.

## Additional file

Additional file 1: Multilingual abstracts in the six official working languages of the United Nations. (PDF 402 kb)

## Abbreviations

AIDS: Acquired immune deficiency syndrome
BMGF: Bill and Melinda Gates Foundation
DALYs: Disability-adjusted life years
EID: Emerging infectious disease
EVD: Ebola virus disease
H7N9: Avian influenza A
IDP: Infectious Disease of Poverty
IDRC: International Development Research Centre
MERS: Middle East respiratory syndrome
MERS-CoV: Middle East respiratory syndrome coronavirus (MERS-CoV)
NTDs: Neglected tropical diseases
OA: Open access
SARS: Severe acute respiratory syndromes
TDR: Special Programme for Research and Training in Tropical Diseases
UN: The United Nations
WHO: The World Health Organization
WOS: Web of science
