# Supplementary material for: InfectiousDiseases ofPoverty, the first five years
Source: Infect Dis Poverty. 2017 May 4;6:96. doi: 10.1186/s40249-017-0310-6 (PMC5415955; doi:10.1186/s40249-017-0310-6)
Supplement: Additional file 1: — Multilingual abstracts in the six official working languages of the United Nations. (PDF 402 kb) [file 40249_2017_310_MOESM1_ESM.pdf]

Translation of the abstract into the six official working languages of the United Nations

الخمس سنوات الأولي لمجلة الأمراض المعدية المرتبطة بالفقر

وي وانغ، جين تشن، هوي-فنغ شنغ، نا-نا وانغ، بين يانغ، شياو-نونغ تشو، روبرت بيرجكويس

الملخص

علي الرغم من تحول التركيز في مجال البحوث الصحية من الأمراض المعدية إلي الأمراض غير المعدية فإن الأمراض المعدية المرتبطة بالفقر تظل عبئا صحيا كبيرا علي المستوي العالمي لذلك فإن هناك حاجة ماسة إلي منصة يتم من خلالها التواصل وتبادل البحوث المتعلقة بهذا النوع من الأمراض وذلك لتسهيل ترجمة المعرفة إلي مشاريع وأدوات فعالة للقضاء علي هذه الأمراض، وإنطلاقا من مبادرة صحة واحدة وعالم واحد تم إنشاء مجلة مفتوحة الوصول تحت مسمي الأمراض المعدية المرتبطة بالفقر وذلك مشاركة بين مركز البحوث الحيوية الطبية (BioMed) والمعهد القومي للأمراض الطفيلية (NIPD) التابع للمركز الصيني للوقاية من الأمراض والسيطرة عليها وذلك في الخامس والعشرين من أكتوبر من عام 2012 وهدفت المجلة إلي تحديد وتقييم البحوث والثغرات المعلوماتية التي تعوق التقدم نحو إيجاد تدخلات جديدة لمشاكل معينة في مجال الصحة العامة في العالم النامي، ومنذ إفتتاح المجلة في 2012 وحتي الأن تم نشر عدد 256 ورقة علمية وبغض النظر عن قلة عدد المقالات الإفتتاحية ومقالات الرأي للمحررين وكذلك الرسائل إلي المحررين فإن العدد الغالب من المنشورات عبارة عن مقالات بحثية شكلت نسبة 69.5٪ ومقالات مرجعية بنسبة 21.5٪ وكان العدد الكلي للناشرين المساهمين هو 1081 ناشر ينتمون لمؤسسات مختلفة موزعة علي عدد 68 من البلدان والأقاليم والمناطق، وتم فهرست المجلة في العديد من قواعد البيانات الطبية الحيوية الرئيسية، بما في ذلك شبكة العلوم ونظام تحليل وإسترجاع المنشورات الطبية علي الانترنت التابع للمكتبة القومية الأمريكية للطب (PubMed) وموقع إسكوبس (Scopus) وقاعدة بيانات أكسيربتا (Embase) وقد تم تعيين أول معامل تأثير للمجلة في عام 2015 وكان مقداره 4.11 والذي أصبح 2.13 في الوقت الحالي، وفي الخمس سنوات الماضية إستقبلت مجلة الأمراض المعدية المرتبطة بالفقر مقالات علمية من 90 بلد وإقليم ومنطقة عبر الست القارات بمعدل قبول سنوي للنشر نسبته أقل من 40٪ وذلك لجميع المساهمات وبين تحليل محتوي المجلة أن الأمراض الإستوائية المهملة (NTDs) متبوعة بالثلاثة أمراض الكبرى (فيروس نقص المناعة البشرية أو الإيدز، المالاريا، السل) ويليهما الأمراض المعدية قد شكلوا جميعا نسبة 88٪ من جميع المنشورات، وبالإضافة إلي ذلك تم نشر عشرة سلاسل من القضايا الموضوعية تحتوي كليا علي 118 مقال علمي نشرت كأجزاء منفصلة عن الخمس أعداد الأولي وقد تم إقتباس هذه المنشورات عدد 975 مرة بمتوسط 8.3 إقتباس لكل منشور، ويتمثل التحدي الحالي للمجلة في تحديد مواضيع البحث المتطورة وجذب ونشر مقالات من الدرجة الأولي مما يؤدي إلى زيادة أهمية وتأثير المجلة في مجال الأمراض المعدية المرتبطة بالفقر.

Translated from English version into Arabic by Mohamed R. Habib

《贫困所致传染病（英文）》五年发展历程

Wei Wang, Jin Chen, Hui-Feng Sheng, Na-Na Wang, Pin Yang, Xiao-Nong Zhou, Robert Bergquist

摘要

虽然卫生研究领域的重点可能从传染病转向慢性非传染性疾病，但贫困所致传染病仍造成严重的全球卫生疾病负担。因此需要一个全球化的平台来沟通和分享贫困所致传染病相关研究成果，以便将其转化为有效的方法和工具来消除这些疾病。2012 年 10 月 25 日，BioMed Central 和中国疾病预防控制中心寄生虫病预防控制所联合创办了《贫困所致传染病（英文）》杂志。该刊立足于“同一健康，同一世界”的理念，致力于确定和评估阻碍发展中国家特定公共卫生问题新干预措施的研究和信息空白领域。自 2012 年 10 月 25 日创刊以来，5 年间该刊共发表 256 篇文章。

除少数的社论（editorial）、观点（opinion）、评述（commentary）和读者来信（letter to the editor）外，该刊发表的主要文章类型是研究论文（69.5%）和辖域综述（21.5%）。256 篇文章的 1 081 位作者分别来自 68 个国家的 323 个机构。该刊被主要国际生物医学数据库收录，包括 Web of Science、PubMed、Scopus 和 Embase。2015 年该刊首获影响因子 4.11。2016 年，第二个影响因子为 2.13。在过去 5 年中，该刊收到来自 6 大洲 90 个国家和地区的来稿，其年度接受率保持在 40%以下。内容分析结果显示，该刊发表被忽视的热带病相关文章最多，其次是“三大疾病”（HIV/AIDS、疟疾和结核病）和一般传染病，共占总发文量的 88%。此外，该刊在前五卷中共发表 10 个专辑，包括 118 篇文章，总被引量为 975，平均被引频次为 8.3。目前，该刊的主要任务是跟踪最前沿的研究课题，吸引并发表一流的文章，逐步提升该刊在其领域的重要性和影响力。

Translated from English version into Chinese by Jin Chen

Maladies Infectieuses de la Pauvreté, les cinq premières années

Wei Wang, Jin Chen, Hui-Feng Sheng, Na-Na Wang, Pin Yang, Xiao-Nong Zhou, Robert Bergquist

Résumé

Bien que l’objectif dans le domaine de la recherche en santé serait en train de passer des maladies infectieuses aux maladies non transmissibles, les maladies infectieuses de la pauvreté demeurent un important fardeau de morbidité de santé mondiale. Une plateforme mondiale de communication et de partage de recherches sur ces maladies est donc impérative afin de faciliter l'application des connaissances en des approches efficaces et outils pour leur élimination. Basée sur sa mission de ‘Un Seul Monde, Une Seule Santé’, une nouvelle revue à accès ouvert, sous l’appellation: Maladies Infectieuses de la Pauvreté (MIP), a été lancée par BioMed Central, en partenariat avec l'Institut National des Maladies Parasitaires (INMP), Centre Chinois de Contrôle et de Prévention des Maladies (China CDC), le 25 Octobre 2012. Son objectif est d'identifier et d'évaluer la recherche et les lacunes en matière d'information qui entravent le progrès vers de nouvelles interventions d’un problème particulier de santé publique dans le tiers monde. Depuis le premier numéro du journal MIP le 25 Octobre 2012, un nombre total de 256 manuscrits ont été publiés au cours des cinq années suivantes. Hormis un petit nombre d'éditoriaux, d'opinions et de lettres à l'éditeur, ses principales publications sont des articles de recherche (69,5%) et des articles de synthèse (21,5%). Un total de 1081 d’auteurs répartis entre 323 affiliations dans 68 pays, territoires et régions ont produit ces 256 publications. Le journal est indexé dans de grandes bases de données biomédicales internationales, y compris le Web of Science, PubMed, Scopus et Embase. En 2015, il lui a été affecté à son premier facteur d’impact (4.11), qui est maintenant 2.13. Au cours des cinq dernières années, il a reçu des manuscrits venant de 90 pays, territoires et régions des six continents avec un taux annuel d'acceptation de tous les articles maintenu à moins de 40%. L'analyse des contenus montre que les Maladies Tropicales Négligées (MTNs), suivies par les ‘Trois Grandes Infections’ à savoir: le VIH/SIDA, le paludisme et la tuberculose, et les maladies infectieuses en général représentent 88% de toutes les publications. En outre, une série de 10 thématiques couvrant 118 publications en tout, a été publié en tant que parties distinctes dans les cinq premiers volumes. Ces publications ont été citées 975

fois, ce qui équivaut à une moyenne de 8,3 fois par publication. Le défi actuel consiste à identifier des thèmes innovants de recherche, à attirer et à faire des publications de premier rang conduisant de plus en plus à revaloriser l'importance et l'impact de la revue dans son domaine.

Translated from English version into French by Kokouvi Kassegne

Инфекционные болезни бедности, в первые пять лет

Wei Wang, Jin Chen, Hui-Feng Sheng, Na-Na Wang, Pin Yang, Xiao-Nong Zhou, Robert Bergquist

Реферат

Хотя основное внимание в области медицинских исследований может быть перенесено с инфекционных на неинфекционные заболевания, инфекционные болезни бедности остаются тяжелым бременем болезни глобальной проблемой здравоохранения. Глобальная платформа для общения и обмена опытом по изучению этих заболеваний необходима для облегчения процесса преобразования знаний в эффективные подходы и инструменты для их ликвидации. На основе миссии “одно здоровье, одна Всемирная ”, новый открытый-доступный журнал , инфекционные заболевание бедности (IDP), были запущены BioMed Central в партнерстве с Национальным Институтом паразитарных заболеваний (NIPD), Китайский центр по контролю и профилактике заболеваний (ЦКЗ Китая), 25 октября 2012 года. Его цель 一 выявить и оценить научно-исследовательские и информационные пробелы, которые препятствуют прогрессу в направлении новых мероприятий для решения конкретной проблемы общественного здравоохранения в развивающихся странах. Итого 1081 соавторов разделили между 323 филиалами по 68 странам, территориям и регионам, которые выпускали эти 256 изданий. Журнал индексируется в крупнейших международных биомедицинских базах данных, включая веб-науки, PubMed, Scopus и Embase. В 2015 году он был назначен его первым импакт-фактором (4.11), который сейчас 2.13. В течение последних пяти лет, IDP получил рукописи из 90 стран, территорий и регионов на шести континентах с годовой ставкой принятия всех взносов поддерживается на уровне менее 40%. Из первого выпуска IDP от 25 октября 2012 года, в общей сложности 256 рукописи были опубликованы в течение следующих пяти лет. Только небольшое количество редакционных материалов и отзыв сообщения и письма в редакцию, преобладающие типы публикаций являются научными статьями (69.5%) и комментариями (21.5%). Контент-анализ показывает, что игнорируемые тропические болезни (NTDs), следующие за “большой тройкой” (ВИЧ/СПИД, малярия и туберкулез) и инфекционными заболеваниями в целом составляют 88% от всех публикаций. Кроме того, серия из 10 тематических вопросов, охватывающих 118 публикаций во всех, были опубликованы в качестве отдельной части первых пяти томов. Эти публикации были приведены в 975 раз, что составляет в среднем 8,3 раза на одну публикацию. Нынешняя задача состоит в том, чтобы выявить передовые исследования темы и привлечь и опубликовать первоклассные публикация, ведущие к увеличению важности и значимости журнала в своей области.

Translated from English version into Russian by Hao-Qi Zhang

Enfermedades Infecciosas de la Pobreza, los primeros cinco años

Wei Wang, Jin Chen, Hui-Feng Sheng, Na-Na Wang, Pin Yang, Xiao-Nong Zhou, Robert Bergquist

Abstracto

A pesar de que el foco de la investigación en el área de la salud pudiese estar cambiando de enfermedades infecciosas a enfermedades no comunicables, las enfermedades infecciosas de la pobreza siguen siendo una gran carga de las enfermedades que preocupan a la salud mundial. Una plataforma global para comunicar y compartir la investigación llevada a cabo sobre estas enfermedades es necesaria para facilitar la traducción del conocimiento en estrategias efectivas y herramientas para su eliminación. Basados en la misión "Una salud, Un mundo", una nueva revista de acceso abierto "*Infectious Diseases of Poverty*" *(Enfermedades Infecciosas de la Pobreza; IDP por sus siglas en inglés),* fue lanzada por la central de BioMed en asociación con el Instituto Nacional de Enfermedades Parasíticas (NIPD, por sus siglas en inglés), Centro de Control de Enfermedades y Prevención de China (China CDC, por sus siglas en inglés) en octubre 25, 2012. Su objetivo es identificar y evaluar vacíos en la investigación e información que obstaculizan el progreso hacia nuevas intervenciones para un problema particular de salud pública en el mundo en desarrollo. Desde el número inaugural de la revista *IDP* en octubre 25, 2012, se han publicado un total de 256 manuscritos durante los siguientes cinco años. Aparte del número pequeño de editoriales y comunicaciones de opinión y de cartas al editor, el tipo predominante de publicaciones son artículos de investigación (69.5%) y revisiones (21.5%). Un total de 1,081 autores contribuyentes divididos entre 323 afiliaciones en 68 países, territorios y regiones produjeron estas 256 publicaciones. La revista está indexada en las principales bases de datos biomédicas internacionales, incluyendo "Web of Science", "PubMed", "Scopus" y "Embase". En 2015, se asignó su primer factor de impacto (4.11), el cual es ahora 2.13. Durante los pasados cinco años, *IDP* ha recibido manuscritos de 90 países, territorios y regiones de los 6 continentes con una tasa de aceptación anual de todas las contribuciones mantenidas en menos del 40%. El análisis de contenido muestra que las enfermedades tropicales desatendidas (NTDs, por sus siglas en inglés), seguido por las "Tres Grandes" (VIH/SIDA, malaria y tuberculosis) y enfermedades infecciosas en general comprenden un 88% de todas las publicaciones. Además, una serie de 10 números temáticos, que abarcaban 118 publicaciones en total, fueron publicados como partes separadas de los primeros cinco volúmenes. Estas publicaciones fueron citadas 975 veces, lo que equivale a un promedio de 8.3 veces por publicación. El desafío actual consiste en identificar temas de investigación de vanguardia y atraer y publicar publicaciones de primer orden que den lugar a una importancia e impacto cada vez mayores de la revista en su campo.

Translated from English version into Spanish by Laura C Vicente Rodriguez
